# Cholera and Ozone

**Published:** 1866-01

**Authors:** T. Moff T


### CHOLERA AND OZONE.

*To the Editor of the Lancet.*—Sir,—In *The Times* of Aug. 26th and 30th, there are two letters on "Cholera and Ozone," in which directions are given for the production of ozone by the use of phosphorus, and the latter is recommended as a disinfectant. I beg to state that for upwards of seventeen years I have used phosphorus for producing ozone, and for five years I have used it as a disinfectant.

In a paper "On the Luminosity of Phosphorus in connection with Atmospheric Conditions," which I read at the Cambridge meeting of the British Association in 1862, I stated that phosphorus was a valuable disinfectant, but that it was effective only during its luminous condition, as ozone is formed only when it is luminous.

The luminosity and non-luminosity of phosphorus are influenced by atmospheric conditions. High pressure, a low degree of temperature, and wind from the north points of the compass are the conditions of non-luminosity; and low pressure, high temperature, and wind from south points of the compass are the conditions of luminosity. The former atmospheric conditions are those of cholera periods, and cholera disappears with the setting in of the latter.

About the 1st of September, 1853, cholera appeared in Newcastle. On the 20th the number of deaths was—cholera, 108; diarrhœa, 10. On the the 19th the south, or ozoniferous, current of the air set in and continued. On the 28th the number of deaths reported in *The Times* was—cholera, 18; diarrhœa, 2. From the 1st of September to the 19th the mean quantity of ozone was scarcely 1-0 of my scale; but from the latter date to the end of the month it ranged from 3 to 8 daily. The epidemic in London, in 1854, disappeared with the setting in of similar atmospheric conditions.

For purifying apartments I use phosphorus in the following way:—I take a quart bottle with a wide mouth, into which I put rather more than half a pint of water; a piece of cork carrying a flat piece of phosphorus with a clean cut surface, floats upon the water. The mouth of the bottle is loosely covered with a cork. The bottle is then placed, first in one part, and then in another, of the apartment to be purified until the peculiar smell of ozone is detected, or until my test papers indicate 1· of my ozone scale. The process of purifying may be performed night and morning, or oftener. For purifying air in the neighborhood of street gratings or in sewers, I simply suspend a piece of phosphorus from the grating. In apartments the temperature may be sufficiently high to keep phosphorus luminous under all atmospheric conditions; but in sewers it will be luminous and non-luminous according to the height of the barometer, the temperature of the surrounding air, and the direction of the wind, and ozone will be produced only when it is luminous. I am, Sir, your obedient servant,

—*London Lancet.* T. MOFFAT, M.D.

